# Perceived coach support and competitive information processing among elite athletes: the mediating role of self-regulation ability

**DOI:** 10.3389/fpsyg.2026.1911170

**Published:** 2026-08-06

**Authors:** WenJun Kou, Libao Cui

**Affiliations:** 1 Zhaoqing University, Zhaoqing, Guangdong, China; 2 Guangzhou Vocational University of Science and Technology, Guangzhou, Guangdong, China

**Keywords:** coach support, competitive information processing, competitive pressure, elite athletes, self-regulation, sport psychology

## Abstract

Elite sport increasingly requires athletes to manage large volumes of competition-relevant information under conditions characterized by uncertainty, time pressure, and continuous adaptation. Although previous research has identified important perceptual, cognitive, and motivational foundations of athletic expertise, less attention has been given to how supportive coaching environments are associated with athletes’ ability to organize and use competition-relevant information. Drawing on Information Scaffolding Theory, this cross-sectional study examined whether perceived coach support is linked to competitive information processing through regulatory information management capability, and whether competitive pressure functions as a boundary condition. Survey data were collected from 500 Chinese competitive athletes from elite university, provincial elite, and national elite sport settings. Structural equation modeling indicated that perceived coach support was positively associated with regulatory information management capability and competitive information processing. Regulatory information management capability showed a positive indirect association between perceived coach support and competitive information processing. Conditional indirect effects were statistically significant at low, mean, and high levels of competitive pressure; however, the index of moderated mediation was not significant, indicating that the hypothesized moderation by competitive pressure was not supported. The findings are consistent with a statistical mediation model in which coach support and self-regulatory information management are linked with competitive information processing. Because the design is cross-sectional and based on self-report data, the results should be interpreted as associative rather than causal.

## 1 Introduction

Elite sport increasingly depends on athletes’ ability to interpret and utilize competition-relevant information under conditions characterized by uncertainty, time pressure, and continuous adaptation. Athletes must identify meaningful cues, distinguish signal from noise, and adjust tactical responses as competitive situations evolve. At elite levels, where differences in physical preparation and technical proficiency are often marginal, information-processing capability may become a critical source of competitive advantage. The central theoretical question is therefore not whether athletes process information, but how some athletes develop the capacity to transform unstable competitive cues into actionable tactical understanding while others remain overwhelmed by the same performance environment. Perceptual-cognitive research has provided important insights into this issue by showing that expert performers tend to exhibit superior visual search patterns, anticipation skills, and attentional control compared with less experienced athletes (Mann et al., 2007; Williams and Ford, 2008; Dong et al., 2024; Kittel et al., 2024). These studies have substantially advanced understanding of the cognitive foundations of expertise, yet they largely conceptualize information processing as an individual accomplishment. Such an explanation remains incomplete because athletes do not learn the meaning of competitive information in isolation. Instead, they acquire, interpret, and refine information within structured training environments where coaches repeatedly shape how performance situations are understood. Through tactical instruction, feedback, video review, and performance correction, athletes learn which cues deserve attention, which patterns indicate opportunity or risk, and how competitive uncertainty should be interpreted. This observation suggests that information processing may be influenced not only by cognitive capacity but also by the social environments in which athletes learn to recognize and organize information. Coaches occupy a particularly influential position within this process because they do more than provide encouragement or emotional support. They help athletes classify situations, prioritize information, direct attention toward diagnostic cues, and develop frameworks for interpreting complex competitive events. Existing research has established that coach support contributes to motivation, confidence, psychological well-being, and performance outcomes, yet considerably less is known about whether supportive coaching environments contribute to the development of athletes’ information-processing capability. As a result, an important theoretical gap remains regarding how externally provided guidance becomes an internally regulated capacity for processing competitive information. Addressing this issue requires moving beyond explanations that focus exclusively on individual cognition and examining the statistical pathways through which social support is associated with competitive information processing. The present study addresses this unresolved problem by investigating how perceived coach support is associated with competitive information processing through regulatory information management capability and how this process varies under conditions of competitive pressure.

### 1.1 Conceptual distinctiveness of competitive information processing

Competitive information processing (CIP) is conceptualized in this study as the sport-specific capability through which athletes acquire, interpret, integrate, update, and apply performance-relevant information under competitive constraints. The concept is intentionally process-oriented because competitive performance depends not only on what athletes know or which option they ultimately select, but also on how they transform unstable environmental cues into tactically meaningful information. Athletes operate within information-rich environments in which signals emerge from opponents, teammates, spatial configurations, score dynamics, bodily feedback, and coach communication. The challenge is rarely the simple presence of information. Rather, it involves determining which cues deserve attention, how those cues should be interpreted, whether their meaning remains valid as competition evolves, and how information should be translated into adaptive action. This focus distinguishes CIP from several established constructs. Decision-making research explains how athletes select among available alternatives, yet it typically begins after relevant information has already been identified and represented (Raab and Johnson, 2007; Low et al., 2023). Tactical intelligence captures sport-specific knowledge structures, strategic understanding, and awareness of game principles, but it provides a less precise account of how athletes manage rapidly changing information under competitive pressure. Situational awareness emphasizes environmental perception, comprehension, and projection (Endsley, 1995), yet offers limited insight into how athletes learn which cues become meaningful within a specific sporting context. Expertise remains indispensable for understanding elite performance broadly (Ericsson et al., 1993; Williams and Ford, 2008; DeCouto et al., 2024), although its conceptual breadth often obscures the specific information-processing mechanisms through which performance advantages emerge. These perspectives have generated important insights, but each addresses only part of the informational challenge athletes encounter during competition.

The need for CIP emerges from a theoretical gap shared across these traditions. Existing frameworks explain how athletes make decisions, what they know, how they perceive environments, or why experts outperform novices, yet they provide less explanation for how socially transmitted guidance becomes individually deployable competitive cognition. Athletes rarely develop information-processing capability independently. Through tactical instruction, feedback, video review, performance analysis, and repeated correction, coaches influence how athletes learn to recognize, prioritize, and interpret competition-relevant information. Over time, these externally provided structures must be internalized and transformed into self-directed processing routines capable of functioning under uncertainty, time pressure, and performance demands. CIP captures this translation process. It occupies the conceptual space between social guidance and competitive cognition by explaining how coaching environments, tactical language, training design, and self-regulatory practices become information-processing capability. Within this framework, competitive information advantage is not treated as a purely individual cognitive trait, nor as a direct consequence of accumulated expertise. Instead, it is understood as a capability that develops through the interaction between socially structured learning experiences and athletes’ ongoing regulation of information use. The value of CIP therefore lies not merely in introducing another performance-related construct, but in providing a conceptual bridge through which social-context theories and perceptual-cognitive theories of elite sport can be more effectively integrated.

### 1.2 Why existing constructs fail to explain the present problem

Existing constructs provide important but incomplete explanations of how athletes manage competitive information. Decision-making research offers a powerful lens because competition ultimately requires action selection. Its contribution lies in explaining how athletes choose among available alternatives and how choice quality varies across expertise, pressure, and task demands (Raab and Johnson, 2007; Low et al., 2023). Yet decision making often begins after the informational foundations of choice have already been established. A pass, shot, defensive adjustment, or pacing change is not selected from raw reality but from an athlete’s representation of the competitive situation. The process through which relevant cues are noticed, interpreted, prioritized, and integrated remains less visible within decision-focused accounts. Tactical intelligence addresses a different aspect of performance by emphasizing strategic knowledge, game understanding, and awareness of tactical principles. This perspective recognizes that athletes rely on accumulated sport-specific knowledge when navigating competition. However, possessing tactical knowledge does not necessarily guarantee effective information handling under rapidly changing conditions. Athletes may understand what should occur within a tactical system while still failing to recognize when unfolding events deviate from expected patterns. Situational awareness moves closer to this challenge by focusing on perception, comprehension, and projection within dynamic environments (Endsley, 1995). Although this framework explains how performers understand competitive situations, it is less explicit about how athletes learn which environmental cues become meaningful and why some cues acquire greater diagnostic value than others. Across these traditions, information processing is often assumed rather than directly theorized. CIP is proposed to address this omission by focusing on the continuous transformation of competitive cues into tactically usable information.

The limitations of existing constructs become particularly visible when examining how coaching environments influence competitive cognition. Sport expertise remains the most comprehensive framework for explaining high-level performance because it encompasses technical skill, perceptual learning, tactical knowledge, physical preparation, and psychological development (Ericsson et al., 1993; Williams and Ford, 2008; DeCouto et al., 2024). Its strength lies in capturing the broad accumulation of performance-related capabilities. At the same time, this breadth makes it difficult to isolate the specific mechanism through which social support contributes to information-processing capability. If all forms of high-level functioning are treated as expertise, the pathway connecting coaching practices to competitive cognition becomes conceptually diffuse. The present study therefore introduces CIP not because existing constructs are inadequate, but because each leaves part of the translation process unexplained. Decision making focuses on selection, tactical intelligence on knowledge, situational awareness on comprehension, and expertise on broad performance development. None directly explains how socially transmitted guidance becomes an athlete’s capacity to process competition-relevant information independently. CIP occupies this conceptual space. It captures how athletes learn to recognize meaningful cues, regulate information use, update interpretations, and transform socially structured learning experiences into autonomous competitive cognition. In this sense, CIP functions as a conceptual bridge linking social-context theories of coaching with perceptual-cognitive theories of elite performance.

### 1.3 The translation problem in elite sport

The central issue addressed in this study is how externally provided coaching support is associated with athletes’ own management of competitive information. Sport psychology has generated substantial evidence showing that coaching environments are related to athlete development and that information-processing capability contributes to performance. What remains less developed is a theory of how socially structured guidance becomes individually regulated information use. The gap is therefore not merely empirical but conceptual. Coach support is typically conceptualized as a social or motivational resource, whereas information-processing ability is often treated as an individual cognitive capability. Information Scaffolding Theory is introduced to connect these domains by focusing on how coaches structure cue selection, interpretation, feedback, and tactical meaning-making in ways that may help athletes organize their own information management.

The present study uses Information Scaffolding Theory as a sport-specific extension of existing coaching and self-regulation perspectives. The theory does not replace perceptual-cognitive expertise, self-determination theory, or ecological approaches to athlete development. Instead, it specifies the informational function of coach support: coaches may reduce informational ambiguity by directing attention toward diagnostic cues, highlighting tactical patterns, and providing interpretive frames through which competition can be understood. Regulatory information management capability is therefore examined as a statistical mediator linking perceived coach support with competitive information processing. This framing is intentionally cautious because the present cross-sectional design can test whether the data are consistent with the proposed theoretical model, but it cannot establish a developmental sequence.

### 1.4 Theoretical positioning

The model developed in this study seeks to refine how competitive information capability is understood in elite sport. Existing research has typically treated information processing as an individual cognitive capability that varies according to expertise, attention, perception, or decision quality. Information Scaffolding Theory extends this view by emphasizing that athletes’ information processing is also embedded in a social learning environment shaped by coaches. The novelty of the theory lies in specifying the link between coach-provided informational structure and athlete-regulated information management. In this account, coach support is not only motivational or relational; it also functions as an informational scaffold that may be associated with how athletes identify, organize, and apply competition-relevant cues.

This perspective also requires a more precise account of the mechanism through which socially structured information becomes athlete-owned capability. Regulatory information management capability is introduced to address this issue. The construct does not refer to personality, self-discipline, or generalized self-control. Instead, it reflects the sport-specific capacity to regulate goals, allocate attention, monitor performance, and maintain information quality under changing competitive demands (Bandura, 1991; Zimmerman, 2000; Carvalho and Araújo, 2022; Morris et al., 2022). Motivational internalization remains relevant because athletes must be willing to engage with coach-provided guidance (Ryan and Deci, 2017; Lemelin et al., 2022; Zhang et al., 2025). However, motivation alone cannot explain how information becomes usable during performance. Athletes must determine which cues deserve attention, whether existing interpretations remain valid, and how information should be updated as competition unfolds. Regulatory information management capability captures this process. It explains how externally provided informational structures become internally regulated routines capable of functioning under pressure. In this way, the model moves beyond asking whether coach support is beneficial and instead specifies how coaching environments contribute to the development of competitive information-processing capability.

## 2 Theory and hypotheses

### 2.1 Coach support as information scaffolding

Traditional explanations of competitive information processing are grounded in perceptual expertise, deliberate practice, and cognitive skill development. These perspectives have substantially advanced understanding of why skilled athletes outperform less experienced performers. Perceptual-expertise research suggests that experts identify meaningful patterns more efficiently, anticipate actions more accurately, and allocate attention more effectively than novices (Mann et al., 2007; Williams and Ford, 2008). Deliberate-practice theory explains how repeated, effortful, and feedback-rich training gradually produces domain-specific performance advantages (Ericsson et al., 1993; Renshaw et al., 2022; Chow and Kee, 2022), while cognitive skill theory describes how complex operations become increasingly organized and efficient through learning (Anderson, 1982; Russo et al., 2022). Collectively, these traditions establish that information advantage is acquired rather than innate. Their limitation lies elsewhere. They explain how athletes become better information processors, but provide less explanation of how the informational environment itself becomes structured before processing occurs. Athletes do not encounter competitive cues as neutral information. Through coaching, feedback, tactical instruction, video review, and training design, they learn which cues deserve attention, which cues are misleading, how patterns should be categorized, and how observations should be connected to future action. Information Scaffolding Theory begins from this premise. It proposes that coach support contributes to competitive information processing by organizing the athlete’s informational environment before information is processed independently. Coaches direct attention toward diagnostic cues, provide interpretive frameworks through which situations can be understood, and assign value to information in relation to tactical goals and performance demands. Supportive coaching therefore serves not only motivational or relational functions but also cognitive-developmental functions by shaping how athletes learn to notice, interpret, and utilize competitive information.

A competing explanation is that athletes with stronger information-processing capability simply receive better coaching because they are more talented, more coachable, or more likely to be selected into advanced developmental systems. This selection perspective is plausible because elite coaches often devote greater resources to athletes who already display exceptional potential. However, selection alone provides a limited explanation for how athletes acquire shared cue vocabularies, sport-specific interpretive categories, and self-monitoring routines. These capabilities emerge through repeated interaction with coaching environments rather than through talent alone. Information Scaffolding Theory offers a stronger explanation because it specifies the cognitive function of support. Coaches externalize informational structures that athletes gradually internalize. A coach may initially direct attention toward a particular cue, encourage interpretation of its tactical meaning, compare judgments with performance feedback, and eventually expect athletes to regulate the same process independently. Through repeated exposure, externally provided informational structures become athlete-owned processing routines. This internalization process represents the critical bridge between coach support and competitive information processing. Effective scaffolding is not dependence on instruction but the gradual development of autonomous information use. The argument is especially relevant in elite sport, where physical and technical differences are often small and performance depends increasingly on the ability to detect subtle changes, update interpretations, and respond adaptively under pressure. Accordingly, coach support should be positively associated with competitive information processing because supportive coaches help construct the informational frameworks through which athletes learn to engage with competition-relevant information.

*H1*: Perceived coach support is positively associated with competitive information processing among elite athletes.

### 2.2 Coach support and regulatory information management capability

The next theoretical question concerns what coach support becomes once it is internalized by the athlete. Traditional explanations often portray support as an external resource that improves performance through instruction, feedback, encouragement, or guidance. Such explanations are useful but incomplete because external support has limited value unless athletes can utilize it when direct intervention is no longer available. Elite performance frequently depends on athletes’ ability to regulate information independently during competition, often in situations where coaches cannot provide immediate correction or direction. The present study therefore shifts attention from support as a resource to support as a developmental process. Regulatory information management capability is introduced to capture this process. Rather than reflecting general self-discipline or a stable personality characteristic, the construct refers to athletes’ capacity to regulate competition-relevant information through goal regulation, attention regulation, and performance monitoring. Goal regulation allows athletes to establish, prioritize, and revise objectives in response to changing performance demands. Attention regulation enables athletes to maintain focus on diagnostic information while resisting distraction. Performance monitoring allows athletes to evaluate whether current actions are producing intended outcomes and to update behavior when necessary. Together, these processes enable athletes to transform external guidance into self-directed information use.

A competing perspective suggests that self-regulation primarily reflects pre-existing individual differences. Athletes may enter elite programs with varying levels of discipline, attentional control, or reflective capacity that emerge from prior experiences, family influences, or personal development. Although this explanation captures part of the picture, it provides a limited account of how sport-specific regulatory routines emerge. General self-discipline does not automatically translate into the ability to monitor tactical execution, regulate attention toward relevant competitive cues, or convert feedback into adaptive performance strategies. These capabilities must be learned within performance environments that repeatedly expose athletes to standards, feedback, and opportunities for guided reflection. Supportive coaches contribute to this process by providing interpretive feedback, encouraging autonomous problem solving, establishing performance expectations, and creating conditions in which athletes can gradually assume responsibility for information management (Jakobsen, 2022; Kim and Choi, 2024; Ricketts et al., 2025). Over time, externally guided regulation becomes regulatory ownership. Athletes learn not only what information matters, but also how to manage that information independently under competitive demands. This developmental process is particularly important in elite sport because performance often depends on athletes’ ability to maintain informational quality when direct coaching input is unavailable. From this perspective, coach support should be understood as an antecedent of regulatory information management capability rather than merely as a source of immediate performance assistance.

*H2*: Perceived coach support is positively associated with regulatory information management capability among elite athletes.

### 2.3 Regulatory information management and competitive information processing

Competitive information processing becomes most vulnerable when the volume of available information exceeds an athlete’s capacity to manage it. Competitive environments rarely suffer from a shortage of cues. Instead, athletes must navigate multiple signals that compete for attention, carry uncertain meaning, and change relevance as situations unfold. Under such conditions, performance depends not only on what athletes know but also on how effectively they regulate the information they encounter. Regulatory information management capability addresses this challenge by helping athletes maintain alignment between performance goals and information use, protecting attention from irrelevant or threat-related cues, and evaluating whether current interpretations remain appropriate as competition evolves (Eysenck et al., 2007; Morris et al., 2022). The central issue is therefore not information availability but information control. Athletes who cannot regulate information effectively may become overwhelmed by the very cues intended to support performance.

An alternative explanation is that competitive information processing depends primarily on perceptual expertise or sport-specific knowledge. This perspective is supported by substantial evidence showing that expert athletes possess richer tactical schemas, more efficient visual search strategies, and superior anticipation skills (Mann et al., 2007; Williams and Ford, 2008). However, expertise alone does not guarantee effective information use. Athletes may recognize relevant patterns yet fail to adapt when conditions change, persist with outdated interpretations, or allocate attention toward cues that are no longer diagnostic. What distinguishes effective information processing is not merely possessing information but maintaining information quality in real time. Regulatory information management capability provides a more precise explanation because it specifies the processes through which information remains useful under competitive demands. Goal regulation establishes informational priorities, attention regulation maintains focus on relevant cues, and performance monitoring enables athletes to evaluate whether existing interpretations remain valid. Together, these processes transform information abundance into tactical clarity. From this perspective, athletes with stronger regulatory information management capability should be better able to detect, interpret, update, and utilize competition-relevant information, resulting in higher levels of competitive information processing.

*H3*: Regulatory information management capability is positively associated with competitive information processing among elite athletes.

### 2.4 Regulatory information management as a statistical mediator

The translation problem becomes most visible when considering how social resources are converted into competitive advantage. Coach support undoubtedly provides athletes with valuable information through instruction, feedback, tactical explanation, and performance review. Yet the presence of information does not guarantee its effective use during competition. Athletes must recognize when information is relevant, maintain attention to it under changing conditions, evaluate whether existing interpretations remain accurate, and update responses as situations evolve. These processes cannot be fully explained by support alone. A direct-support perspective assumes that coaches influence performance primarily because they provide better guidance or more accurate information. Although plausible, this explanation becomes less convincing in competitive environments where athletes must act independently and where opportunities for direct coaching intervention are limited. Information that remains dependent on external guidance cannot fully function as competitive advantage. The critical question is therefore not whether coaches provide information, but how coach-provided information becomes usable when coaches are no longer directing action in real time.

The present study examines regulatory information management capability as the proposed statistical mediator. Information scaffolding initially exists outside the athlete through the coach’s efforts to organize attention, define meaningful cues, and structure interpretation. The present model proposes that coach support is associated with competitive information processing partly because athletes who perceive stronger informational support also report greater capacity to regulate goals, allocate attention, monitor feedback, and maintain information quality. This pathway is theoretically directional, but the empirical evidence should be interpreted as an indirect association because all focal variables were measured in a cross-sectional survey.

*H4*: Regulatory information management capability mediates the association between perceived coach support and competitive information processing.

### 2.5 Why competitive pressure matters

Competitive pressure is not merely a contextual characteristic of elite sport. It represents a condition under which the quality of information processing becomes increasingly difficult to maintain. As performance consequences become more salient, athletes must continue interpreting, updating, and applying competition-relevant information while simultaneously managing heightened arousal, uncertainty, time constraints, and evaluative demands. Pressure therefore creates a fundamental cognitive challenge. The information environment often becomes more consequential at precisely the moment it becomes more difficult to manage. Attentional Control Theory suggests that pressure increases the likelihood that task-relevant processing will be disrupted by threat-related concerns, self-evaluative thoughts, and competing attentional demands (Eysenck et al., 2007). Under these conditions, athletes must preserve informational accuracy despite distractions that threaten the quality of cue detection, interpretation, and decision preparation. The theoretical importance of pressure lies not simply in its presence, but in its ability to alter the cognitive conditions under which information is processed.

Alternative boundary conditions such as age, gender, training history, or sport type may explain differences between athletes, but they provide less insight into when regulatory capability becomes most consequential. Competitive pressure directly changes the demands placed on information processing itself. As uncertainty increases and informational windows become narrower, athletes can no longer rely solely on stable routines or previously established interpretations. They must continuously maintain informational priorities, resist distraction, detect meaningful changes, and revise tactical understanding as situations evolve. Under relatively low-pressure conditions, even imperfect regulatory systems may be sufficient because the informational environment remains comparatively stable. Under high-pressure conditions, however, failures of information management become increasingly costly. The influence of regulatory information management capability should therefore become stronger as competitive pressure increases because athletes with superior regulatory capacity are better able to preserve information quality when performance demands intensify. This perspective positions pressure not as a demographic or contextual control variable, but as a theoretically meaningful condition that determines when the pathway from social support to competitive information advantage becomes most visible.

*H5*: Competitive pressure moderates the association between regulatory information management capability and competitive information processing, such that the association is stronger when competitive pressure is higher. Consequently, the indirect association between coach support and competitive information processing through regulatory information management capability is expected to be stronger under higher competitive pressure.

## 3 Method

### 3.1 Participants and procedure

The study sample consisted of 500 Chinese competitive athletes recruited from multiple structured sport settings. In this manuscript, the term elite athletes is used operationally rather than as a claim that all participants competed at the same professional or international level. Participants were classified as elite for the purposes of the study if they were actively involved in organized competitive training, had at least 2 years of competitive sport experience, trained regularly under coach supervision, and competed at elite university, provincial elite, or national elite levels. The sample included 126 elite university athletes, 212 provincial elite athletes, and 162 national elite athletes. This operational definition was selected because the study focuses on coach-led competitive information processing among athletes exposed to formal training, competition preparation, and performance evaluation. At the same time, the breadth of this definition is a limitation, and competitive level was examined as a descriptive sample characteristic and as a robustness consideration.

Eligibility criteria were established to ensure that participants possessed sufficient competitive experience for the constructs examined in this study. Athletes were required to have completed at least 2 years of organized competitive training, to be actively participating in structured sport programs, to have regular contact with a primary coach, and to have recent competition experience. Participants were recruited through sport programs and coach-facilitated distribution of the survey link. Participation was voluntary, and respondents were informed that their answers would be used only for research purposes and would not be shared with coaches in individually identifiable form. Responses were screened for completeness, response-pattern irregularities, and eligibility consistency before analysis. Cases with substantial missing data on the focal constructs or invalid response patterns were excluded before the final analytic sample was established. Missing values within retained cases were minimal and were handled through the estimation procedures described in the analysis section.

### 3.2 Measures

Construct development followed a theory-driven measurement strategy. Items were generated or adapted to reflect the conceptual definitions of the focal constructs rather than to maximize empirical overlap among measures. Perceived coach support was adapted from established coach-athlete relationship and autonomy-supportive coaching literature, whereas competitive information processing and regulatory information management capability were developed for the present sport-information context. For adapted items, wording was reviewed to ensure conceptual equivalence with the source construct while fitting Chinese competitive sport settings. For newly developed items, item generation was guided by the definitions of cue acquisition, interpretation, integration, updating, goal regulation, attentional allocation, feedback monitoring, and information-quality control. The questionnaire was reviewed by sport psychology and coaching-domain experts for content relevance, clarity, and redundancy before administration. Items were also checked for athlete readability and contextual appropriateness. Because several measures are adapted or newly specified, the measurement evidence reported here should be regarded as preliminary and should be extended through independent validation, longitudinal measurement invariance testing, and criterion-related validation in future research.

Because the survey was administered to Chinese competitive athletes, the adapted English-language items were translated into Chinese and then back-translated into English by bilingual researchers familiar with sport psychology terminology. Discrepancies between the original and back-translated versions were discussed and resolved by the research team, with emphasis on preserving the meaning of coaching support, regulatory information management, competitive information processing, and competitive pressure in Chinese sport contexts. Before formal data collection, the questionnaire was pilot tested for readability, item clarity, response burden, and contextual relevance with competitive athletes who were not included in the final analytic sample. Feedback from this pilot check was used to simplify ambiguous wording and remove redundant expressions while preserving the intended construct coverage.

Perceived coach support was assessed using six items adapted from the coach-athlete relationship and self-determination literature (Mageau and Vallerand, 2003; Jowett and Ntoumanis, 2004; Stephen et al., 2022; Zhao and Jowett, 2023). Items focused on athletes’ perceptions that coaches provided meaningful guidance, helped organize tactical information, encouraged reflection, and connected training experiences to competitive performance. A representative item was, “My coach helps me understand which performance cues are most important.” Regulatory information management capability was measured using six items derived from social-cognitive and metacognitive perspectives on self-regulation (Bandura, 1991; Zimmerman, 2000; Carvalho and Araújo, 2022). Items captured goal regulation, attentional control, and performance monitoring during competition. A representative item was, “I monitor whether my tactical choices are working during competition.” Competitive information processing was measured using seven items informed by perceptual-cognitive expertise, situational awareness, and sport decision-making research (Endsley, 1995; Mann et al., 2007; Kittel et al., 2024). Items assessed athletes’ ability to identify relevant cues, integrate information sources, update interpretations, and translate information into tactical action. A representative item was, “I quickly notice changes in opponents’ tactical patterns.” Competitive pressure was assessed using four items reflecting perceived demands associated with time pressure, performance consequences, and high-stakes competition. A representative item was, “I frequently need to make decisions under time and score pressure.” All multi-item constructs were scored using mean values, with higher scores indicating higher levels of the corresponding construct. Age, training history, weekly training volume, and prior injury experience were included as control variables. A short marker-attention scale was incorporated to support common-method bias assessment.

### 3.3 Data analysis

Data analysis proceeded in several stages. Descriptive statistics were first examined to characterize the sample and study variables. Internal consistency and convergent validity were evaluated using Cronbach’s alpha, composite reliability, average variance extracted, and standardized factor loadings. Discriminant validity was evaluated using HTMT ratios and confirmatory factor analysis model comparisons. Confirmatory factor analysis and structural equation modeling were conducted in R using the lavaan package with maximum-likelihood estimation. The structural model was estimated with standardized path coefficients reported as *β* values. HC3 robust standard errors were used in the conditional process estimates to reduce sensitivity to heteroscedasticity. Bootstrap confidence intervals with 5,000 resamples were used for indirect associations. Before interpreting the structural model, the data were screened for missingness, response irregularities, approximate normality, multicollinearity, and influential observations. Variance inflation factors were below conventional concern thresholds, and the measurement model showed acceptable fit before structural paths were interpreted. Because the study used a cross-sectional self-report design, mediation and moderated mediation results are described as statistical indirect associations rather than evidence of causal or developmental mechanisms. Because the sample included elite university, provincial elite, and national elite athletes, supplementary robustness models also included competition level as a set of dummy-coded covariates, with elite university athletes as the reference group. These models were used to examine whether the focal associations remained substantively similar after accounting for competitive level.

## 4 Results

The results are reported in four steps: sample description, measurement quality, associations among the focal constructs, and tests of the statistical mediation and moderated mediation model. Because the study is cross-sectional, the reported paths are interpreted as associations rather than evidence of temporal or causal effects.

Table 1 presents the demographic and sport-related characteristics of the sample. Participants represented multiple sport contexts and competitive levels, providing variability in performance demands while maintaining a common exposure to structured coaching environments. On average, athletes reported more than 8 years of training experience and nearly 19 hours of weekly training, indicating sustained involvement in organized competitive sport. These characteristics suggest that the sample possessed substantial experience with coach-led development, tactical preparation, and formal competition, making it appropriate for examining the relationships among perceived coach support, regulatory information management capability, competitive pressure, and competitive information processing.

**Table 1:** Demographic and sport-related characteristics of the sample.

Variable	Category	Value
Age	Mean (SD)	23.05 (2.72)
Training years	Mean (SD)	8.49 (2.78)
Weekly training hours	Mean (SD)	18.94 (5.10)
Gender	Female	190
Gender	Male	310
Sport type	Combat sport	121
Sport type	Individual endurance sport	125
Sport type	Skill or precision sport	89
Sport type	Team ball sport	165
Competition level	Elite university	126
Competition level	National elite	162
Competition level	Provincial elite	212

Values are counts unless otherwise indicated.

Table 2 presents the descriptive statistics for the focal constructs. Mean scores indicate moderate-to-high levels of perceived coach support, regulatory information management capability, and competitive information processing among the athletes. Competitive pressure was reported at a comparatively lower level, although the average score remained above the scale midpoint. Standard deviations suggest adequate variability across all constructs, supporting subsequent multivariate analyses. The observed distributions indicate that the measures captured meaningful differences in athletes’ perceptions and experiences, providing an appropriate basis for the reliability, validity, and hypothesis-testing procedures that follow.

**Table 2:** Descriptive statistics of the focal constructs.

Variable	*M*	SD	Min	Max
Perceived coach support	3.46	0.85	1.17	5.00
Self-regulation ability	3.50	0.83	1.17	5.00
Competitive information processing	3.47	0.82	1.14	5.00
Competitive pressure	3.20	0.92	1.00	5.00

All focal constructs were scored from 1 to 5.

Table 3 presents the reliability and convergent validity results for the study constructs. Cronbach’s alpha values ranged from 0.815 to 0.876, while composite reliability values ranged from 0.878 to 0.904, exceeding recommended thresholds and indicating satisfactory internal consistency. Average variance extracted values ranged from 0.573 to 0.643, supporting adequate convergent validity across all constructs. The marker attention scale also showed acceptable measurement quality and was retained for common-method bias assessment. Overall, the results provide evidence that the study measures possess satisfactory reliability and convergent validity, supporting their use in subsequent analyses.

**Table 3:** Reliability and convergent validity assessment.

Construct	Items	Cronbach’s *α*	CR	AVE
Perceived coach support	6	0.861	0.896	0.590
Self-regulation ability	6	0.868	0.901	0.604
Competitive information processing	7	0.876	0.904	0.573
Competitive pressure	4	0.815	0.878	0.643
Marker attention	3	0.703	0.835	0.628

CR, composite reliability; AVE, average variance extracted.

Table 4 presents the correlations among the focal constructs. Perceived coach support was positively associated with regulatory information management capability and competitive information processing. Regulatory information management capability was also positively associated with competitive information processing. These correlations were moderate in magnitude, suggesting meaningful relationships among the constructs while remaining below levels that would raise concerns regarding construct redundancy. Competitive pressure showed relatively weak associations with the other variables, indicating that it represents a distinct aspect of the competitive environment. Overall, the correlation pattern provides preliminary support for the proposed theoretical model and supports further multivariate analyses.

**Table 4:** Correlations among the study variables.

Variable	Coach support	Self-regulation	CIP	Competitive pressure
Coach support	1.000	0.480***	0.377***	0.054
Self-regulation	0.480***	1.000	0.424***	0.072
CIP	0.377***	0.424***	1.000	0.108*
Competitive pressure	0.054	0.072	0.108*	1.000

CIP, competitive information processing. Correlations are Pearson correlations. *p < 0.05; **p < 0.01; ***p < 0.001.

Table 5 presents the heterotrait-monotrait (HTMT) ratios used to assess discriminant validity. All HTMT values were below the recommended threshold of 0.85, providing evidence that the study constructs are empirically distinct. The highest HTMT value was observed between regulatory information management capability and competitive information processing (0.486), but this value remained well below established cutoffs. The results therefore support adequate discriminant validity and suggest that the focal constructs capture related yet conceptually distinct aspects of athletes’ competitive experiences.

**Table 5:** Discriminant validity assessment using HTMT ratios.

Construct	Perceived coach support	Self-regulation ability	Competitive information processing	Competitive pressure	Marker attention
Perceived coach support	1.000	0.554	0.434	0.072	0.041
Self-regulation ability	0.554	1.000	0.486	0.090	0.105
Competitive information processing	0.434	0.486	1.000	0.130	0.086
Competitive pressure	0.072	0.090	0.130	1.000	0.048
Marker attention	0.041	0.105	0.086	0.048	1.000

HTMT values below 0.85 are preferred.

Table 6 presents the results of the confirmatory factor analyses. The hypothesized five-factor model showed satisfactory fit to the data, with all fit indices meeting commonly recommended standards. The alternative models in which perceived coach support and regulatory information management capability were combined, or regulatory information management capability and competitive information processing were combined, showed substantially poorer fit. The single-factor model performed considerably worse than all multi-factor solutions. These findings provide evidence that the focal constructs are empirically distinguishable and support the proposed measurement structure.

**Table 6:** Confirmatory factor analysis model comparisons.

Model	*χ* ^2^	d*f*	*χ*^2^/d*f*	CFI	TLI	RMSEA	SRMR
Hypothesized five-factor model	518.41	289	1.79	0.953	0.944	0.054	0.057
Merged coach support and self-regulation	778.41	293	2.66	0.904	0.891	0.081	0.077
Merged self-regulation and CIP	833.41	293	2.84	0.893	0.881	0.085	0.082
Single-factor model	1418.41	299	4.74	0.782	0.761	0.124	0.119

CFI, comparative fit index; TLI, Tucker-Lewis index; RMSEA, root mean square error of approximation; SRMR, standardized root mean square residual.

Table 7 summarizes the common method bias diagnostics. The Harman single-factor test indicated that the first factor accounted for 26.8% of the total item variance, remaining below commonly used thresholds. Marker-variable correlations with the focal constructs were low, suggesting limited shared method variance. The common latent factor analysis also produced only minimal changes in model fit indices. Collectively, these findings suggest that common method bias is unlikely to represent a dominant source of variance in the data, although the possibility of some shared method effects cannot be completely excluded.

**Table 7:** Common method bias diagnostics.

Test	Result	Decision
Harman single-factor diagnostic	First factor = 26.8% of item variance	Below 50% rule-of-thumb threshold
Marker-variable correlation	Highest marker-to-core construct *r* = 0.082	Low shared method signal
Common latent factor comparison	ΔCFI = 0.008; ΔRMSEA = 0.003	No dominant common-method factor indicated

Diagnostics reduce but do not eliminate common-method concerns.

Table 8 presents the path estimates for the hypothesized statistical model. Coach support was positively associated with competitive information processing in the total-effect model, providing support for H1. Coach support was also positively associated with regulatory information management capability, supporting H2. Regulatory information management capability was positively associated with competitive information processing, supporting H3. The indirect association from coach support to competitive information processing through regulatory information management capability supported H4 as a statistical mediation pattern. Competitive pressure was positively associated with competitive information processing, but the interaction between regulatory information management capability and competitive pressure was small and not statistically significant. Thus, the moderation component of H5 was not supported.

**Table 8:** Path estimates for the conditional process model.

Outcome	Predictor	*β*	SE	*p*	Sig.	95% CI
CIP total-effect model	Coach support	0.372	0.044	<0.001	***	[0.285, 0.459]
CIP total-effect model	Competitive pressure	0.088	0.040	*p* = 0.029	*	[0.009, 0.167]
Self-regulation	Coach support	0.477	0.038	<0.001	***	[0.402, 0.553]
Self-regulation	Competitive pressure	0.047	0.037	*p* = 0.212		[−0.027, 0.120]
CIP conditional process model	Coach support	0.224	0.049	<0.001	***	[0.128, 0.320]
CIP conditional process model	Self regulation	0.313	0.047	< 0.001	***	[0.221, 0.404]
CIP conditional process model	Competitive pressure	0.076	0.039	*p* = 0.052		[−0.001, 0.153]
CIP conditional process model	Self-regulation × pressure	0.039	0.040	*p* = 0.329		[−0.039, 0.117]

HC3 robust standard errors. CIP, competitive information processing.

Table 9 presents the bootstrap estimates of the conditional indirect effects. The indirect association between perceived coach support and competitive information processing through regulatory information management capability was statistically significant at low, mean, and high levels of competitive pressure, as all confidence intervals excluded zero. However, the index of moderated mediation included zero, indicating that the size of the indirect association did not differ significantly across levels of competitive pressure. Therefore, H5 was not supported. These results should be interpreted as evidence of a robust indirect association rather than evidence that competitive pressure significantly moderates the indirect pathway.

**Table 9:** Conditional indirect effects and moderated mediation analysis.

Indirect path	Estimate	Boot SE	95% CI lower	95% CI upper	Result
Coach support → self-regulation → CIP at low competitive pressure (−1 SD)	0.131	0.031	0.071	0.192	Supported
Coach support → self-regulation → CIP at mean competitive pressure	0.149	0.025	0.102	0.201	Supported
Coach support → self-regulation → CIP at high competitive pressure (+1 SD)	0.168	0.032	0.107	0.232	Supported
Index of moderated mediation	0.019	0.019	−0.020	0.055	Exploratory; not conclusive

Bootstrap confidence intervals use 5,000 resamples.

Table 10 presents the effect size and predictive relevance statistics for the focal relationships. Coach support showed a medium-to-large association with regulatory information management capability, whereas regulatory information management capability showed a meaningful effect on competitive information processing. The direct effect of coach support on competitive information processing was comparatively smaller, consistent with the partial mediation pattern observed in the conditional process analyses. The interaction between regulatory information management capability and competitive pressure produced a negligible effect size. Cross-validated *Q*^2^ values were positive across the models, indicating acceptable predictive relevance. Collectively, these findings suggest that the focal relationships are not only statistically significant but also possess practical relevance within the proposed theoretical framework.

**Table 10:** Size and predictive relevance statistics.

Outcome	Focal predictor	*R*^2^ full	*f* ^2^	*Q* ^2^	Interpretation
Self-regulation	Coach support	0.232	0.303	0.212	Medium-to-large practical effect
CIP	Self-regulation	0.226	0.097	0.186	Medium practical effect
CIP	Coach support direct path	0.226	0.050	0.186	Small direct effect
CIP	Self-regulation × pressure	0.226	0.002	0.186	Small exploratory conditional effect

*f*^2^ values are incremental effect sizes. *Q*^2^ values are five-fold cross-validated predictive relevance estimates.

As an additional robustness check, the focal paths were re-estimated with competition level entered as dummy-coded covariates. The main pattern remained substantively unchanged: coach support remained positively associated with regulatory information management capability (*β* = 0.477), regulatory information management capability remained positively associated with competitive information processing (*β* = 0.310), and the direct association from coach support to competitive information processing remained positive (*β* = 0.226). The interaction between regulatory information management capability and competitive pressure remained non-significant (*β* = 0.038), confirming that the lack of support for H5 was not explained by omission of competition level.

## 5 Discussion

### 5.1 Solving the translation problem

The central contribution of the findings is not that a causal mediation pathway was established, but that the data are consistent with an indirect association linking perceived coach support, regulatory information management capability, and competitive information processing. Although perceived coach support was positively associated with competitive information processing, the theoretically important pattern lies in the association through regulatory information management capability. Athletes who perceived stronger coach support also reported stronger capacity to organize goals, allocate attention, monitor feedback, and preserve information quality, and this regulatory capability was in turn associated with stronger competitive information processing. Because the evidence is cross-sectional, the findings should be understood as support for the plausibility of Information Scaffolding Theory rather than as proof of a developmental process.

### 5.2 Competitive advantage is socially constructed

A second theoretical contribution of the study is the more cautious reconceptualization of competitive information capability as both individually regulated and socially embedded. Research on elite performance has traditionally emphasized physical preparation, technical proficiency, perceptual-cognitive expertise, and deliberate practice as primary foundations of competitive superiority. These perspectives remain essential. The present findings add that perceived coach support is associated with athletes’ self-reported ability to manage competition-relevant information, suggesting that informational features of the coach-athlete environment deserve greater attention in sport psychology and coaching research.

### 5.3 Coaches as architects of information environments

A third theoretical contribution of the present study concerns the role of coaching in the development of competitive information processing. Research in sport psychology has traditionally emphasized the motivational, emotional, and relational functions of coach support, highlighting its influence on athlete confidence, commitment, satisfaction, and psychological well-being. Although these functions remain important, the present findings suggest that they do not fully explain how coaching contributes to competitive adaptation. The results support a broader interpretation in which coaches function as architects of athletes’ information environments. Rather than merely encouraging effort or maintaining positive relationships, coaches help determine where athletes direct attention, which cues are treated as diagnostically meaningful, how ambiguous situations are interpreted, and how performance feedback is transformed into future action. This perspective helps explain why coach support remained positively associated with competitive information processing even after regulatory information management capability was introduced into the model. The influence of coaching appears to extend beyond affective support and may operate through the construction of informational structures that shape how athletes understand competition itself. This distinction is theoretically important because not all forms of support are equally relevant to information-processing capability. Praise may strengthen confidence without improving cue selection, instruction may increase compliance without fostering independent interpretation, and emotional closeness may enhance trust without necessarily improving tactical adaptation. By contrast, information-oriented support organizes attention, interpretation, feedback, and reflection in ways that athletes can later apply without direct coach intervention. Coaches help athletes learn which environmental signals deserve attention, why particular cues matter, how tactical patterns should be categorized, and how current information should be integrated with prior competitive knowledge. Over time, these externally provided informational frameworks can become internalized through repeated training, competition, feedback, and self-reflection. The developmental value of coaching therefore lies not only in providing support but also in structuring learning experiences that teach athletes how to identify, evaluate, and use performance-relevant information independently. From this perspective, coaching quality should be assessed not only by whether athletes feel supported, motivated, or confident, but also by whether coaching practices cultivate athletes’ capacity to process information autonomously, interpret uncertainty effectively, and adapt to changing competitive demands without continuous external guidance. The present findings suggest that competitive information advantage is partly rooted in this process of informational architecture building, whereby coaches create the conditions under which athletes gradually acquire ownership over the information-processing routines that support elite performance.

### 5.4 Self-regulation as information ownership

A fourth theoretical contribution of the present study is the reconceptualization of self-regulation as a mechanism of information ownership rather than merely a desirable individual characteristic. In much of the sport psychology literature, self-regulation is typically discussed as a positive psychological resource that helps athletes maintain effort, persist through challenges, and pursue performance goals. The present findings suggest a more specific and theoretically consequential role. Regulatory information management capability appears to function as the mechanism through which externally provided informational structures become athlete-owned competitive capabilities. Coach support can organize attention, highlight diagnostic cues, provide interpretive frameworks, and create meaningful feedback structures, but these resources have limited value unless athletes can regulate their own use of information during competition. Goal regulation allows athletes to transform coaching input into prioritized performance objectives, attention regulation enables athletes to maintain focus on task-relevant cues while filtering distraction, and performance monitoring allows athletes to evaluate whether current interpretations remain useful as competitive situations evolve. This conversion process helps explain why regulatory information management capability mediated the association between coach support and competitive information processing. The findings indicate that coaching does not directly create information advantage; rather, coaching creates conditions under which athletes can develop the capacity to manage information independently. This interpretation also helps distinguish regulatory information management capability from alternative explanatory mechanisms. Motivation may explain why athletes invest effort in training and competition, but it does not directly explain how athletes identify, prioritize, and update competitive information. Confidence may encourage decisive action, yet confident athletes can still attend to irrelevant cues, maintain inaccurate tactical assumptions, or fail to revise interpretations when circumstances change. Regulatory information management capability provides a more precise explanation because it directly concerns the management of information itself. The developmental significance of this mechanism lies in its ability to transform support into autonomy. Athletes who rely on coaches to interpret every cue remain dependent on external guidance, whereas athletes who internalize regulatory routines can carry those informational frameworks into new competitive environments, adapt them to unfamiliar opponents, and continuously refine them through self-monitoring and adjustment. Self-regulation therefore represents more than a positive psychological trait. It is the internal structure that converts socially scaffolded information into autonomous competitive cognition and allows athletes to maintain effective information processing when direct coach intervention is no longer possible. From this perspective, the indirect effect identified in the present study reflects a broader developmental process in which external informational support gradually becomes internalized as athlete-owned regulatory capability, ultimately producing sustainable competitive information advantage.

### 5.5 Why pressure reveals regulatory quality

Competitive pressure remains theoretically relevant because it represents a performance context in which effective information management may become increasingly valuable. However, the present study did not find support for the hypothesized moderating effect of competitive pressure. Although the conditional indirect associations were statistically significant at low, mean, and high pressure levels, the index of moderated mediation was not significant. One interpretation is that regulatory information management capability may be broadly useful across pressure conditions rather than becoming substantially more important only under high pressure. A second possibility is that the competitive pressure measure captured general perceived pressure rather than momentary, event-specific pressure during actual competition. A third possibility is that pressure effects are nonlinear or sport-specific, with different patterns across team, endurance, combat, and skill-based sports. Future studies should examine pressure using longitudinal, diary, experimental, or competition-proximal designs and should test whether objective competition stakes or physiological arousal provide stronger boundary conditions.

### 5.6 Toward an ecological theory of competitive information processing

The present study also contributes an integrative theoretical framework for understanding competitive information processing in elite sport. Existing theoretical perspectives each explain important aspects of athlete development, yet none independently provides a complete explanation for how competitive information-processing capability emerges. Self-determination theory explains how supportive coaching environments facilitate internalization and self-regulated functioning by helping athletes adopt externally provided standards and goals as their own. Attention control theory explains why regulation becomes important when competitive demands threaten goal-directed processing and increase susceptibility to distraction. Information scaffolding theory explains how coaches organize informational environments by directing attention toward meaningful cues, providing interpretive frameworks, and structuring feedback processes that support learning. Each perspective captures a different component of the developmental process, but each also leaves important questions unresolved. Self-determination theory explains motivation and internalization but offers limited explanation for how competitive information itself becomes organized and managed. Attention control theory explains the regulation of attention under demanding conditions but does not address how cue meaning is socially acquired. Information scaffolding theory explains the social origins of learning but requires a regulatory mechanism to explain how externally provided structures become independent athlete capabilities. Integrating these perspectives provides a more comprehensive account of competitive information processing. Coaches create informational architectures that help athletes identify, interpret, and prioritize performance-relevant cues. Athletes gradually internalize these externally structured informational frameworks through regulatory information management capability, transforming coach-provided guidance into athlete-owned routines for goal regulation, attention regulation, and performance monitoring. Competitive demands provide a context in which the effectiveness of these regulatory capacities becomes particularly relevant, although the present findings suggest that their value may extend beyond specific pressure conditions. This integrated perspective represents the central theoretical position of the study. Competitive information processing is neither purely cognitive nor purely social. It is cognitive because athletes must continuously detect, interpret, update, and apply information. It is social because the meaning of competitive information is learned within coaching and training environments. It is regulatory because athletes must independently manage information quality as competitive situations evolve. The model therefore moves beyond simple associations among variables and proposes a layered explanation of elite adaptation in which social scaffolding shapes informational architectures, regulatory capability internalizes those architectures, and competitive performance reflects the effectiveness of the resulting information-processing system.

### 5.7 Practical implications for coaching and training design

The practical message is not simply that coaches should be supportive. It is that support should change the informational work athletes learn to do. Coaches can begin by auditing the specificity of their feedback: instead of telling athletes only what was good or poor, they should name the cue, explain why it mattered, and ask the athlete to connect that cue to the next tactical option. In daily practice, this means using guided questioning, short video clips with cue-stopping points, opponent-pattern worksheets, and debrief prompts such as, “What did you see first?,” “Which cue changed your decision?,” and “What would you monitor next time?” These behaviors turn support into information scaffolding rather than general encouragement. Recent work on coach-athlete relationships and autonomy-supportive coaching similarly suggests that support is most useful when it strengthens athletes’ ownership of learning rather than keeping interpretation in the coach’s hands (Jowett, 2024; Lemelin et al., 2022; Zhang et al., 2025; Zhao and Jowett, 2023). The coach’s role, therefore, is not to provide more information, but to teach athletes how to sort, test, and update information independently.

Training design should also give athletes repeated opportunities to practice regulatory information management under controlled difficulty. A useful progression is to begin with low-noise cue recognition, move to constrained tactical choices, and then add time pressure, score pressure, fatigue, opponent deception, and evaluative consequences. Practice tasks can require athletes to state a pre-performance cue plan, execute under variable information, and complete a short post-action review of whether their attention remained aligned with the plan. Simulation, extended-reality tasks, and representative practice designs may be especially useful when they preserve the perceptual and temporal demands of competition while still allowing coaches to pause, question, and recalibrate athletes’ information use (Kittel et al., 2024; Renshaw et al., 2022; Low et al., 2023). This is the practical “so what” of the model: coach support should be converted into routines that athletes can carry into competition when coaches are no longer able to interpret the situation for them.

Sport organizations can operationalize these ideas by embedding information-scaffolding behaviors into coach education, performance review, and athlete development plans. Coach evaluation should not assess only relationship quality or athlete satisfaction; it should also examine whether coaches help athletes become better at cue selection, tactical explanation, pressure adaptation, and reflective monitoring. Athlete support teams can coordinate video analysts, sport psychologists, and coaches around common cue-language so that athletes receive coherent informational architectures rather than disconnected advice. This is also a mental-health issue, because environments that combine support, psychological safety, and clear role communication may reduce burnout risk while strengthening performance learning (Glandorf et al., 2022; Gerber et al., 2024; Prior et al., 2024; Simpson et al., 2023; Şenel et al., 2025; Woods et al., 2025). The applied goal is concrete: coaches should ask better questions, design better information constraints, and gradually transfer interpretive responsibility to athletes.

### 5.8 Limitations and future research

Several limitations should be considered when interpreting the findings. First, the study used a cross-sectional design, so the proposed model should be interpreted as associative rather than causal. Athletes with stronger competitive information processing may attract more sophisticated coaching support, while athletes with stronger regulatory capabilities may evaluate coaching environments more positively. Longitudinal, experimental, and intervention-based designs are therefore needed to test temporal ordering and causal direction. Second, all focal variables were measured using self-report questionnaires, which creates risks of common method variance, social desirability, and shared response tendencies. The common method diagnostics reduce but do not eliminate these concerns. Third, several measures, especially competitive information processing and regulatory information management capability, were adapted or newly specified for the present context. The reliability, CFA, HTMT, and model-comparison evidence supports their use in this sample, but the instruments should be treated as preliminary and require further validation. Fourth, the operational definition of elite athletes was broad and included elite university, provincial elite, and national elite athletes. Competitive level may shape coach support, self-regulation, pressure, and information processing, and future work should test the model separately across competitive levels or include competition level as a formal covariate where appropriate.

Additional limitations concern contextual generalizability and the broader ecology of competitive information processing. The sample included Chinese competitive athletes from multiple sport categories, improving breadth but limiting direct generalization to non-Chinese sport systems, professional leagues, youth academies, or national-team contexts outside China. The informational challenges faced by team-sport athletes may differ from those faced by endurance, combat, or skill-based athletes. Future studies should examine sport-specific pathways, compare competition levels, include coach- or observer-rated information-processing outcomes, and use competition-proximal measures of pressure. Such designs would help determine whether Information Scaffolding Theory generalizes across cultural, institutional, and sport-specific contexts.

## 6 Conclusion

The present study examined how perceived coach support, regulatory information management capability, competitive pressure, and competitive information processing are associated within elite sport. The findings suggest that competitive information capability is not solely an individual cognitive resource, nor is it simply a by-product of supportive coaching environments. Instead, the cross-sectional evidence is consistent with a model in which perceived coach support is linked to competitive information processing partly through athletes’ self-reported regulatory information management capability. Competitive pressure remained theoretically relevant but did not significantly moderate the indirect pathway in this sample. The findings therefore support a cautious interpretation: Information Scaffolding Theory offers a useful framework for studying how coach support and athlete self-regulation are associated with competitive information processing, but longitudinal, experimental, and competition-proximal research is needed before stronger developmental or causal claims can be made.

## Data Availability

The original contributions presented in the study are included in the article/Supplementary material, further inquiries can be directed to the corresponding author.
